# Chitosan‐Orange (*Citrus sinensis*) Essential Oil Coatings With Combined Treatment of KMnO_4_ to Enhance the Shelf‐Life and Storage Quality of Ercolini Pears

**DOI:** 10.1002/fsn3.70045

**Published:** 2025-03-10

**Authors:** Yasir Abbas Shah, Saurabh Bhatia, Ahmed Al‐Harrasi, Mohammad Tarahi, Mahbubur Rahman Khan

**Affiliations:** ^1^ Natural and Medical Sciences Research Center University of Nizwa Nizwa Oman; ^2^ School of Health Science University of Petroleum and Energy Studies Dehradun India; ^3^ Department of Food Science and Technology, School of Agriculture Shiraz University Shiraz Iran; ^4^ Department of Food Processing and Preservation Hajee Mohammad Danesh Science & Technology University Dinajpur Bangladesh

**Keywords:** biopolymer coating, fruit preservation, natural preservative, postharvest treatment

## Abstract

The study evaluated the effects of chitosan coating combined with Orange ( *Citrus sinensis* ) essential oil (OEO) and potassium permanganate (KMnO_4_) sachets as ethylene inhibitors on maintaining quality and extending the shelf‐life of Ercolini pears. Additionally, it examined the reduction of superficial scalding in pears stored at 25°C ± 2°C. for 14 days. Different parameters of the fruits were evaluated during the storage period which include weight reduction, incidence of superficial scald, surface microstructure (SEM), water contact angle, total phenolic content (TPC), total flavonoid content (TFC), antioxidant activity, and sensory attributes. The group of fruits treated with the chitosan‐OEO coating and KMnO_4_ exhibited the lowest weight reduction (3.76%) and the lowest incidence of superficial scald compared to the control. SEM analysis showed that the treated pears had smoother surface microstructures, indicating effective contact between the coating and the fruit tissue. The water contact angle increased to 100.15° for the chitosan‐OEO‐coated fruits, indicating enhanced water barrier properties. The highest levels of TPC, TFC, and antioxidant activity were observed in the chitosan‐OEO‐coated fruit group at the end of the storage period. Sensory analysis revealed that the chitosan‐OEO‐coated pears, combined with ethylene scavengers, scored higher in appearance, texture, flavor, mouthfeel, aroma, and overall acceptability compared to the control. This treatment method demonstrates promising potential for extending the shelf‐life of pears while preserving their visual and nutritional quality.

## Graphical abstract

The study evaluated the effects of chitosan coating combined with Orange ( *Citrus sinensis* ) essential oil (OEO) and potassium permanganate (KMnO_4_) sachets as ethylene inhibitors on maintaining quality and extending the shelf‐life of Ercolini pears. The group of fruits treated with the chitosan‐OEO coating and KMnO_4_ exhibited the lowest weight reduction and lowest incidence of superficial scald compared to the control. The highest levels of TPC, TFC, and antioxidant activity were observed in the chitosan‐OEO‐coated fruit group at the end of the storage period.

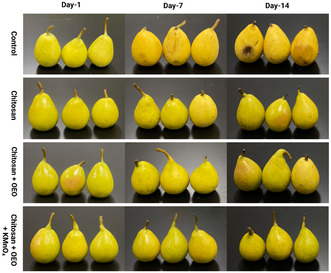

## 1 Introduction

The increasing global population presents a challenge in ensuring worldwide food security and achieving sustainable production of nutritious, high‐quality food. Fruits and vegetables, crucial for promoting human health and combating malnutrition, require the application of appropriate post‐harvest technologies to preserve their quality (Romanazzi and Moumni 2022). Pears have a limited storage capacity and are particularly susceptible to adverse conditions during harvesting, transportation, and reloading processes (Pathare and Al‐Dairi 2021). Throughout the interval spanning from the time of harvesting to consumption, pears experience a range of physiological and biochemical alterations. Some of these changes are advantageous for enhancing quality, including improvements in aroma and taste, the breakdown of starch into more digestible sugars, and a decrease in levels of organic acids. On the other hand, the increased rates of transpiration and respiration after harvest lead to inevitable deterioration resulting in subsequent losses (Saquet 2019). The superficial scald also referred to as storage scald, is a physiologically relevant storage disorder common in apples and pears. It is characterized by the development of brown or black patches on the skin of the fruits. However, the symptoms of scald become apparent when the fruits are exposed outside of storage, thus limiting their market suitability (Whitaker et al. 2009). Therefore, adequate storage and post‐harvest treatment are essential in improving the quality of the pears.

It has been reported that edible coatings offer excellent results in enhancing the storage quality of fruits and vegetables (Pandey et al. 2022). Chitosan is a linear polysaccharide known for its strong antimicrobial and antifungal activity and it is highly effective in reducing fruit decay. Considering the significant properties of chitosan, it has been extensively used as a coating material to extend the storage shelf‐life of a wide range of postharvest fruits and vegetables (Shiekh et al. 2013; Xing et al. 2016). Essential oils are used in coating applications due to their antioxidant and antimicrobial properties and are highly effective in maintaining fruit and vegetable quality and extending the shelf‐life (El‐Mesallamy et al. 2012; Ribeiro‐Santos et al. 2017; Sharma et al. 2021). Several EOs are considered as “GRAS” (generally recognized as safe) by the US Food and Drug Administration due to their favorable safety profiles and considerable antioxidant and antimicrobial effects (Raut and Karuppayil 2014).

Orange essential oil (OEO) obtained from *Citrus sinensis* is reported to offer substantial antioxidant and antimicrobial properties, and it serves as a promising natural source in prolonging the shelf‐life of food products by preventing both oxidation and microbial spoilage (Manzur et al. 2023). It has been reported that one of the major factors causing superficial scald in pears is the oxidation of (E,E)‐α‐farnesene (Torres et al. 2021). Consequently, a natural oil like OEO could serve as an excellent antioxidant material in coatings to delay the development of scald in pears.

Furthermore, ethylene also plays a prominent role in the development of scald by stimulating the synthesis of α‐farnesene (Torres et al. 2021). It is reported that the concentration of ethylene present in the storage atmosphere is directly proportional to the rate of quality deterioration of a wide range of fruits and vegetables (Bower et al. 2003). Potassium permanganate (KMnO_4_) is a powerful ethylene absorbent widely used in fresh fruits and vegetables to delay post‐harvest ripening (Álvarez‐Hernández et al. 2019). Hence, KMnO_4_ sachets alongside chitosan coatings infused with OEO present a promising approach for suppressing scald formation in pears while enhancing the shelf‐life and quality of the fruits. The current study aims to investigate the impact of chitosan coating infused with OEO, along with KMnO_4_ sachets as ethylene inhibitors, on the quality and shelf‐life of pears (cv. Ercolini) stored at room temperature for 14 days.

## 2 Materials and Methodology

### 2.1 Materials

Pears (cv. Ercolini) were purchased from a local market in Nizwa, Oman, 24 h before treatments. The supplier confirmed that the pears underwent no prior treatments. The selection criteria for fruits included uniform size, absence of skin damage, and physiological maturity. Subsequently, the fruits were washed with distilled water and were subsequently air‐dried at ambient temperature to remove surface moisture. Orange ( *Citrus sinensis* ) essential oil (Batch No: NNIWOEO/667/0821) was purchased from Nature Natural India. Chitosan (Low MW, extrapure, 10–150 m. Pas, 90% DA) was purchased from Sisco Research Laboratories (SRL) Pvt. Ltd., Mumbai (India). Acetic acid was procured from Sigma‐Aldrich, while glycerol (99.0% purity) was provided by BDH Laboratory Supplies, London, UK.

### 2.2 Preparation of the Coating Solution and Treatment

Chitosan solution (2% w/v) was prepared by dissolving 2 g of chitosan in 1% acetic acid solution and subjected to magnetic stirring for 3 h. Glycerol (1% v/v) was added to the chitosan solution as a plasticizer to enhance the flexibility and strength of the coating solution. After preparation, the chitosan solution was divided into separate beakers. The fruits were categorized into four groups (each group containing 30 fruits), denoted as COP1‐COP4, with COP1 serving as the control group without any coating. The COP2 group underwent coating by dipping the fruits in the prepared chitosan solution for 30 s, while the chitosan solutions for the COP3 and COP4 groups were supplemented with OEO (1% v/v) and subjected to agitation. Subsequently, the COP3 and COP4 groups were coated with the OEO‐loaded solution and allowed to dry for 2 h. After drying, the pears were carefully packed in sterile plastic zipper bags with small holes made for ventilation and were then stored at 25°C ± 2°C and a relative humidity of 70%. A high gas‐permeable sachet containing 8 g of KMnO4 as an ethylene scavenger was placed inside the packaging, positioned near the pears, in the COP4 group. The fruit groups were evaluated on days 1, 7, and 14.

### 2.3 Appearance Changes

Pears were photographed using a digital camera (iPhone 12 Pro Max, with a 12‐megapixel camera featuring a 2.5× zoom) to monitor changes in appearance at day 0 and weekly for up to 14 days. The same angles and distances were maintained for all photographic documentation.

### 2.4 Weight Loss Measurement

Fruits from each treatment were selected, tagged, and weighed on day 0. Subsequently, the tagged fruits were weighed at 7‐day intervals using a digital electronic balance, with measurements taken in triplicates. The weight loss differences after storage, compared to the initial weight, were expressed as percentages (Lin et al. 2017).

### 2.5 Superficial Scolding Incidence

In compliance with the methodology outlined by Zanella (2003), the rate of superficial scald occurrences was evaluated on days 0, 7, and 14 at 25°C ± 2°C and relative humidity of 70%. The incidence rate was calculated using Equation (1):

(1) $$\text{Incidence rate}\;\left(\%\right)=\frac{\text{Number of fruits affected}}{\text{Total number of fruits}}\times 100$$

### 2.6 Morphological Properties

To generate SEM images, one fruit from each treatment was randomly selected and processed within 24 h after the coating dried. Small sections of the peel were dissected from the equatorial area of the fruit, affixed to a stub, and coated with a thin layer of gold 10 semi‐trained panelists (male, age range 20–35 years old) were recruited for the evaluation of sensory attributes (Khorram et al. 2017). Imaging of the samples was performed using a scanning electron microscope (JSM6510LA, Jeol, Japan). Digital SEM images were then captured to reveal both surface and cross‐sectional morphology, illustrating the pear surface with the applied coating.

### 2.7 Surface Hydrophobicity

Water contact angle (WCA) measurements were taken utilizing a goniometer (OCA 11, Data Physics, Germany) as per the methodology of (Cerqueira et al. 2014). WCA measurements were performed using 1000 μL disposable syringes (DS‐D 1000 SF silicone‐free and solvent‐resistant) and dosing needles (SNS‐D 051/025). The contact angle on fruit surfaces was determined via the sessile drop technique, with measurements completed within less than 30 s.

### 2.8 Total Phenolic Compounds and Total Flavonoid Content

3 g of pear tissue (comprising both the coated surface and pulp) from each sample was taken and underwent homogenization in a solution consisting of methanol and water (80:20, v/v), with a volume of 20 mL. For each analysis, the experiment was conducted in triplicates by taking three different extracts from each group (COP1‐COP4). The homogenized samples were subjected to shaking for 15 min, followed by sonication (Daihan ultrasonic cleaner WUC‐D06H) for 15 min, and then centrifuged (Centrifuge, Frontier 5000 Series Multi Pro, FC5718R) for 10 min at 14,000 RPM and 18,620 RCF. The resultant supernatant was collected, filtered, and stored at low temperatures until further analysis.

The determination of total phenolic content was carried out using the Folin–Ciocalteu colorimetric method (Abdelhameed et al. 2021; Salama et al. 2022; Singleton et al. 1999), with gallic acid utilized as the reference standard for calibration. Findings are presented as gallic acid equivalents (GAE) per 100 g of fresh weight (FW). The quantification of overall flavonoid content was carried out by diluting the extract with aluminum chloride (AlCl_3_) and potassium acetate (CH_3_CO_2_K) as outlined in the methodology by Lin and Tang (2007). Quercetin was used as a standard and the total flavonoid content was presented as mg/100 g FW.

### 2.9 Antioxidant Activity

The DPPH assay was conducted following the procedural guidelines outlined by Brand‐Williams et al. (1995). 0.3 mL of fruit extract was combined with 0.5 mL of the DPPH solution. This mixture was then incubated in darkness for 60 min. The alterations in color were assessed by measuring absorbance at 517 nm. The antioxidant activity of the fruit samples was measured in triplicates for each group by taking three different extracts. The antioxidant activities of the samples were expressed as the percentage inhibition of DPPH radicals.

### 2.10 Sensory Analysis

The sensory attributes of pears were evaluated on days 1, 7, and 14 of storage as per the procedure described in a previous study (Ghafoor et al. 2022). The sensory assessment involved a panel consisting of individuals affiliated with the Natural and Medical Sciences Research Center (NMSRC), (seven males, and three females, aged between18 and 35 years old), were recruited and trained according tothe sensory practices. Evaluated sensory characteristics included appearance, texture, flavor, mouthfeel, aroma, and overall acceptability. Ratings were conducted using a 9‐point hedonic scale, with scores ranging from 1 (Dislike extremely) to 9 (Like extremely) (Kemp et al. 2011).

### 2.11 Statistical Analysis

To assess the significance of deviations among mean values, analysis of variance (ANOVA) was applied, followed by a subsequent post hoc Fisher's test. This statistical methodology was utilized to quantify the significance of variations in the means, with a confidence level established at 95%.

## 3 Results and Discussion

### 3.1 Visual Analysis

**FIGURE 1:**
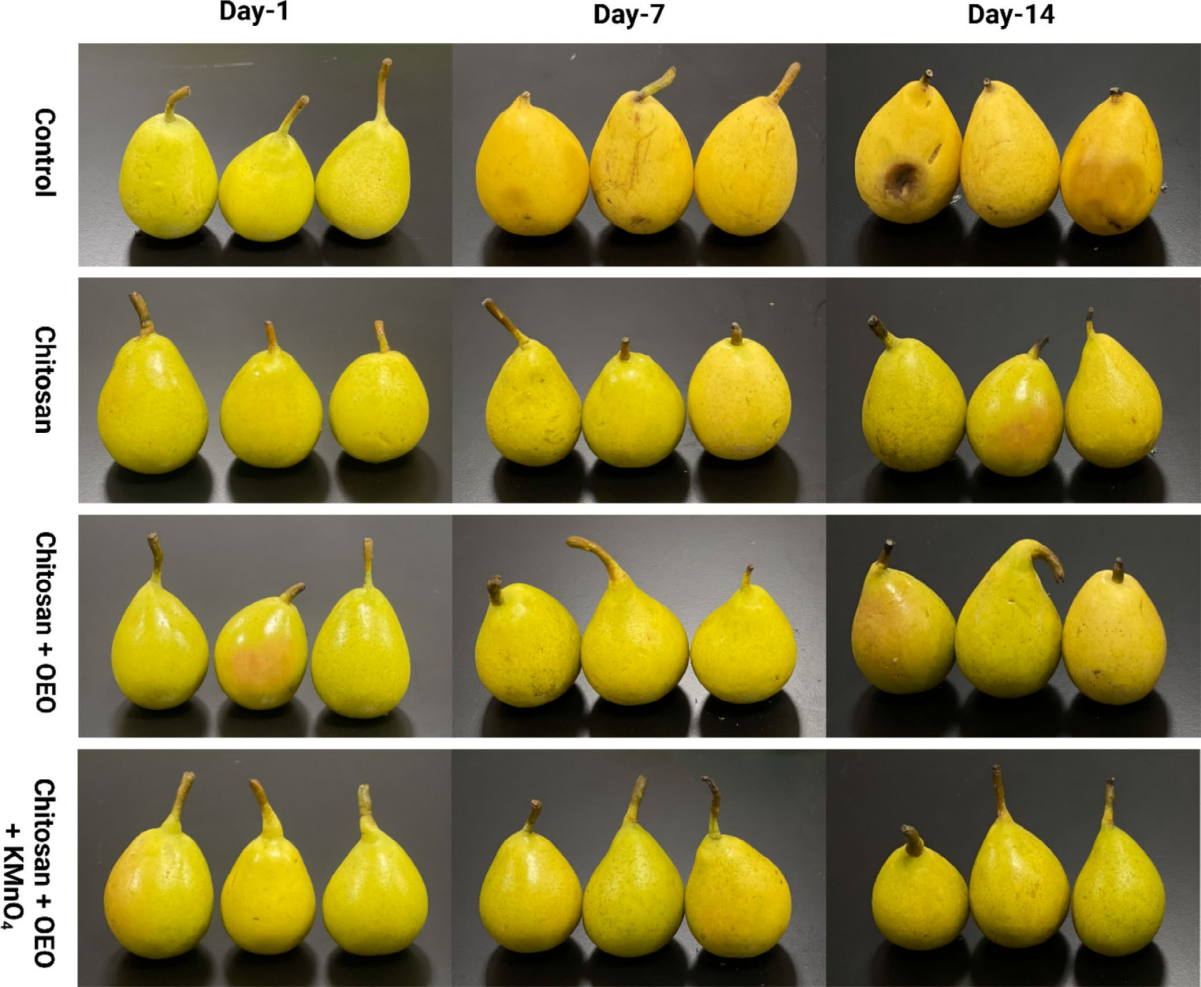
Visual analysis of the fruit samples with different treatments during storage.

The control group (COP1) and coated fruits (COP2‐COP4) with different treatments were subjected to the same storage conditions for 14 days, during which their visual attributes were monitored over the storage period, as illustrated in Figure 1. The COP1 group exhibited no apparent decay until the 4th day of storage, However, after 1 week, the fruits softened, and a noticeable shift in color from green to yellow was observed. On the 9th day of storage, fruits in the control group exhibited dark brown patches and showed a maximum shift in color change due to accelerated ripening, making them unsuitable for the market due to shrinkage, excessive softness, and fungal overgrowth. The fruit groups with coatings (COP2‐COP4) exhibited a shiny surface and retained their green color for up to 14 days during storage, with no evidence of decay detected in any of the coated fruit samples.

The COP2 group coated with chitosan without the incorporation of orange EO showed a slight color change from green to yellow and softening in some fruits was also observed. The COP2 group, treated with chitosan without the addition of orange EO, exhibited a slight shift in coloration from green to yellow. This change was accompanied by noticeable softening in certain fruits on the 12th day of storage. The COP3 fruit group, coated with chitosan and orange essential oil, did not seem different from the COP4 fruit group, which had the same coating with an additional treatment of KMnO_4_ as an ethylene scavenger.

### 3.2 Weight Loss During Storage

**FIGURE 2:**
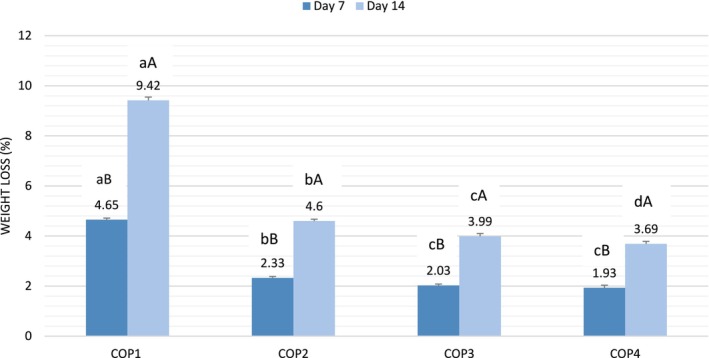
Weight loss (%) of the fruit samples with different treatments during the storage. Values within the same day, followed by different lowercase letters (a–d) are significantly different (*p* < 0.05). Values between days for the same coating followed by different uppercase letters (A, B) indicate a significant difference (*p* < 0.05).

Weight loss is a critical parameter during the storage of coated fruits as it directly impacts their shelf‐life and quality. Excessive weight loss can lead to dehydration and shriveling, decreasing the appeal and nutritional value of the fruit. In the current study, weight loss increased in both the control and coated pear fruits following 14 days of storage. However, the weight loss was notably greater in the control group compared to the coated fruits, as illustrated in Figure 2. During a 14‐day storage period, the control fruit samples exhibited a weight reduction of 9.14%, followed by COP2, COP3, and COP4. The COP4 fruit samples, coated with a chitosan solution loaded with OEO and subjected to additional KMnO_4_ treatment as an ethylene inhibitor, exhibited the minimum weight reduction (3.76%).

It has been reported that polysaccharide‐derived edible coatings act as barriers to gases and moisture, thereby slowing down respiration and dehydration processes. This results in reduced moisture loss from the fruit surface, ultimately preventing overall weight loss of the fruit (Guillén et al. 2013; Khaliq et al. 2016). Chitosan forms a semi‐permeable barrier, effectively controlling gas exchange while reducing water loss via transpiration (Aparicio‐García et al. 2021). The incorporation of EO in chitosan is an effective approach to create a barrier against moisture movement, thereby helping to control the weight loss of fruits. Different studies carried out with the use of chitosan coating loaded with EO revealed a positive effect on the weight loss and quality of fruits. Coating China jujube with chitosan incorporated with cinnamon oil significantly reduced the weight loss of the fruits during storage (Xing et al. 2015). Perdones et al. (2016) demonstrated that strawberries coated with chitosan‐lemon essential oil maintained better quality and exhibited reduced microbial growth during storage. Furthermore, It has been reported that coatings containing essential oils like thyme, oregano, and clove exhibited strong antioxidant activities, which helped preserve the phenolic content and vitamin levels in fruits (Valencia‐Chamorro et al. 2011).

### 3.3 Superficial Scalding Index

The superficial scalding index is a measure used to evaluate the severity of superficial scald, a physiological disorder affecting the skin of certain fruits, most notably apples and pears, during storage. Table 1 summarizes the incidence rates of superficial scald in these groups.

**TABLE 1:** Incidence rate (%) indicating the number of affected fruits as compared to the total number of fruits.

Sample	Day 1	Day 7	Day 14
COP1	0	15%	40%
COP2	0	5%	15%
COP3	0	0	10%
COP4	0	0	10%

COP1: control sample (pears without any coating); COP2: pears coated with 2% w/v chitosan; COP3: pears coated with 2% w/v chitosan and 1% v/v orange essential oil; COP4: pears coated with 2% w/v chitosan, 1% v/v orange essential oil and 8 g KMnO_4_.

The COP1 control group, without any coating, resulted in high superficial scald incidence, reaching 15% on day 7 and 40% on day 14. A high incidence rate demonstrates the susceptibility of untreated fruits to superficial scald, probably because there is no protective barrier to avoid oxidation and ethylene effect. In the case of chitosan‐coated fruits, COP2, superficial scald incidence was lower than that of the control, with 5% affected on day 7 and 15% affected on day 14. The reduced rates of respiration and water loss have likely led to the prevention of the development of scald symptoms. This result aligns with findings from previous studies indicating that chitosan coatings can effectively reduce post‐harvest physiological disorders in fruits (Hossain and Iqbal 2016; Jianglian and Shaoying 2013; Sultana et al. 2019).

Adding OEO to the chitosan coating significantly decreased the incidence of superficial scald. By day 7, no scald was observed. The antimicrobial and antioxidant properties of OEO were likely to enhance the protective effect of chitosan coating. This combination seems to inhibit the oxidative processes responsible for superficial scald, which is in agreement with other investigations in which essential oils have been shown effective in preserving the quality of fruit (Pandey et al. 2022; Rico Rodríguez et al. 2015; Sharma et al. 2016). On the other hand, the addition of an ethylene scavenger (KMnO_4_) to the COP4 group did not show improvement over the COP3 group without significance; both displayed a 10% incidence rate by day 14. This suggests that while the chitosan‐OEO coating effectively reduced scald, the inclusion of an ethylene scavenger might not provide additional benefits under the given conditions.

### 3.4 Total Phenolic and Flavonoid Compounds

Total phenolic content (TPC) is an essential parameter in assessing the quality of fruits during storage, as these compounds contribute to both the nutritional and sensory characteristics of the final products. According to Table 2, the TPC of fruit samples tended to decrease during storage from day 1 to 14, which may be attributed to the breakdown of cell structure as fruits mature (Yousuf et al. 2021). However, chitosan coatings, particularly those containing OEO and KMnO_4_, can significantly help in retaining the phenolic compounds of pears during storage at room temperature. At the end of storage, the lowest TPC was determined in the control group (28.80 ± 0.10 mg GAE/100 g FW), while the highest TPC was observed in COP4‐coated pear fruits (37.40 ± 0.30 mg GAE/100 g FW). These results could be related to the nature of OEO, which can interact with the polymeric matrix of chitosan through hydrogen bonds, facilitating the slow and controlled release of their phenolic compounds into the surrounding medium (Nair et al. 2020). Additionally, blank chitosan coatings have been reported for their ability to enhance the production of phenolic substances by increasing the activity of some specific enzymes, such as phenylalanine ammonia lyase (PAL) (Tokatlı and Demirdöven 2021). Almost similar findings were reported by Popescu et al. (2022), in this study, researchers treated strawberry and apple fruits with chitosan‐based coatings containing 2% ascorbic acid and 7.5% grape seed EO during storage at 4°C and 8°C for 7 days. On the other hand, the use of KMnO_4_ showed promising results in our study to maintain the TFC of pear fruits during storage. This phenomenon suggests the vital role of KMnO_4_ in preserving phenolic compounds by suppressing ethylene production and slowing down the ripening process.

**TABLE 2:** TPC and TFC of the fruit samples with different treatments during storage on days 1, 7, and 14.

Analyses	Samples	Storage days		
		1	7	14
TPC (mg GAE/100 g FW)	COP1	33.85 ± 0.74^bA^	32.56 ± 0.75^cB^	28.80 ± 0.10^dC^
	COP2	35.40 ± 0.06^bA^	33.64 ± 0.76^bcB^	31.81 ± 1.22^cC^
	COP3	35.93 ± 0.26^bA^	35.40 ± 0.06^bA^	34.52 ± 1.43^bA^
	COP4	41.16 ± 2.54^aA^	39.26 ± 2.05^aB^	37.40 ± 0.30^aC^
TFC (mg/100 g)	COP1	9.64 ± 1.72^aA^	8.33 ± 0.57^aB^	7.49 ± 0.59^bC^
	COP2	9.92 ± 0.35^aA^	8.78 ± 1.19^aAB^	8.03 ± 0.90^abB^
	COP3	10.88 ± 0.61^aA^	9.73 ± 1.37^aA^	9.84 ± 0.56^aA^
	COP4	10.03 ± 0.66^aA^	9.87 ± 1.15^aA^	9.82 ± 1.50^aA^

*Note:* Values in the same column followed by different letters are significantly different. Values in the same row (A–C) show significant differences between the days. COP1: control sample (pears without any coating); COP2: pears coated with 2% w/v chitosan; COP3: pears coated with 2% w/v chitosan and 1% v/v orange essential oil; COP4: pears coated with 2% w/v chitosan, 1% v/v orange essential oil and 8 g KMnO_4_; TPC: total phenolic content; TFC: total flavonoid content.

The measurement of total flavonoid content (TFC) in fruit coatings during storage is another important factor for assessing antioxidant activity, maintaining fruit quality, evaluating coating efficacy, and ensuring the nutritional value of the coated fruits. Similar to the results of TPC analysis, the TFC of control and coated pear fruits was gradually decreased during storage at room temperature (Table 2). At day 14, the coated fruits showed higher TFC, ranging from 8.03 to 9.84 mg/100 g, compared to the control sample (7.49 mg/100 g), whereas the chitosan, OEO, and KMnO_4_ treatments did not exhibit significant differences (*p* < 0.05). This may be due to the presence of high amounts of flavonoids, especially hesperidin and neohesperidin, in chitosan‐based OEO biopolymers (Dikmetas et al. 2024; Zhang et al. 2023a). On the other hand, Bal (2018) demonstrated that treating nectarines ( *Prunus persica* cv. Bayramiç Beyazı) with 7 and 10 g KMnO_4_ was the most effective way to maintain the TFC of the fruits during storage at 0°C–1°C for 40 days, due to its effects on delaying the ripening process in the treated fruits.

### 3.5 Antioxidant Activity

**FIGURE 3:**
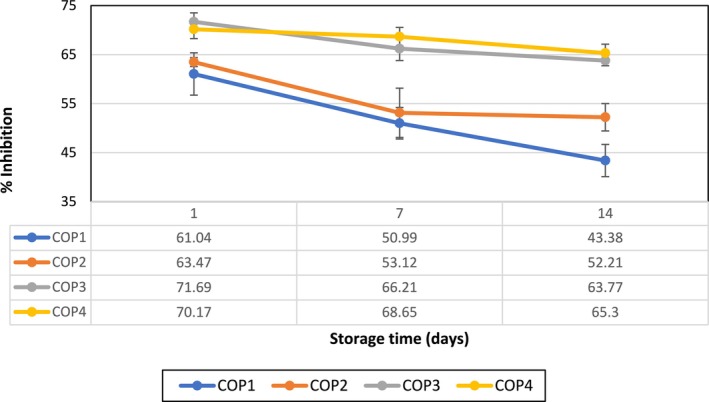
Antioxidant activity (% inhibition of DPPH radical) of the fruit samples with different treatments during the storage.

During post‐harvest storage, oxidative reactions in fruits can lead to the deterioration of their nutritional value, color, flavor, and overall appearance. By assessing the antioxidant activity, it becomes possible to measure the effect of coatings in inhibiting these undesired reactions, thus preserving the quality of the fruits over time (Bhatia et al. 2024). On the other hand, EOs contain various antioxidants, including phenolic compounds and flavonoids, which can help prevent food spoilage and oxidation. These natural preservatives can act synergistically against free radicals, contributing to the overall stability of fruits during storage. Flavonoids are known for their ability to scavenge free radicals and quench oxidative reactions, thereby protecting the fruits from deterioration, while phenolic acids generally act as antioxidants by trapping free radicals (Bhatia et al. 2023; Nair et al. 2020; Oikeh et al. 2020). In the present study, the antioxidant activity of the control and coated samples was determined during 14 days of storage using the DPPH radical scavenging method, and then, the relevant values were expressed as the percentage inhibition of DPPH radicals, as illustrated in Figure 3. It was found that the antioxidant activity of all fruit samples significantly decreased (*p* < 0.05) during storage, which is consistent with our previous results (Table 2). However, the COP3 and COP4 samples represented the highest antioxidant activity in all storage days. This indicates the promising antioxidant activity of chitosan‐based coatings incorporated with 1% v/v OEO and/or 8 g KMnO_4_, which can be attributed to the presence of various bioactive compounds in OEO, mainly limonene and β‐myrcene (Akarca and Sevik 2021), and/or the absorption of ethylene by KMnO_4_, which can maintain higher antioxidants in fruits during storage (Bal 2018). These observations are in line with previous studies on the incorporation of chitosan (Shiri et al. 2013), EO (Iftikhar et al. 2022), or KMnO_4_ (Shaukat et al. 2023) to improve the antioxidant activity of the treated fruit samples.

### 3.6 SEM Analysis

**FIGURE 4:**
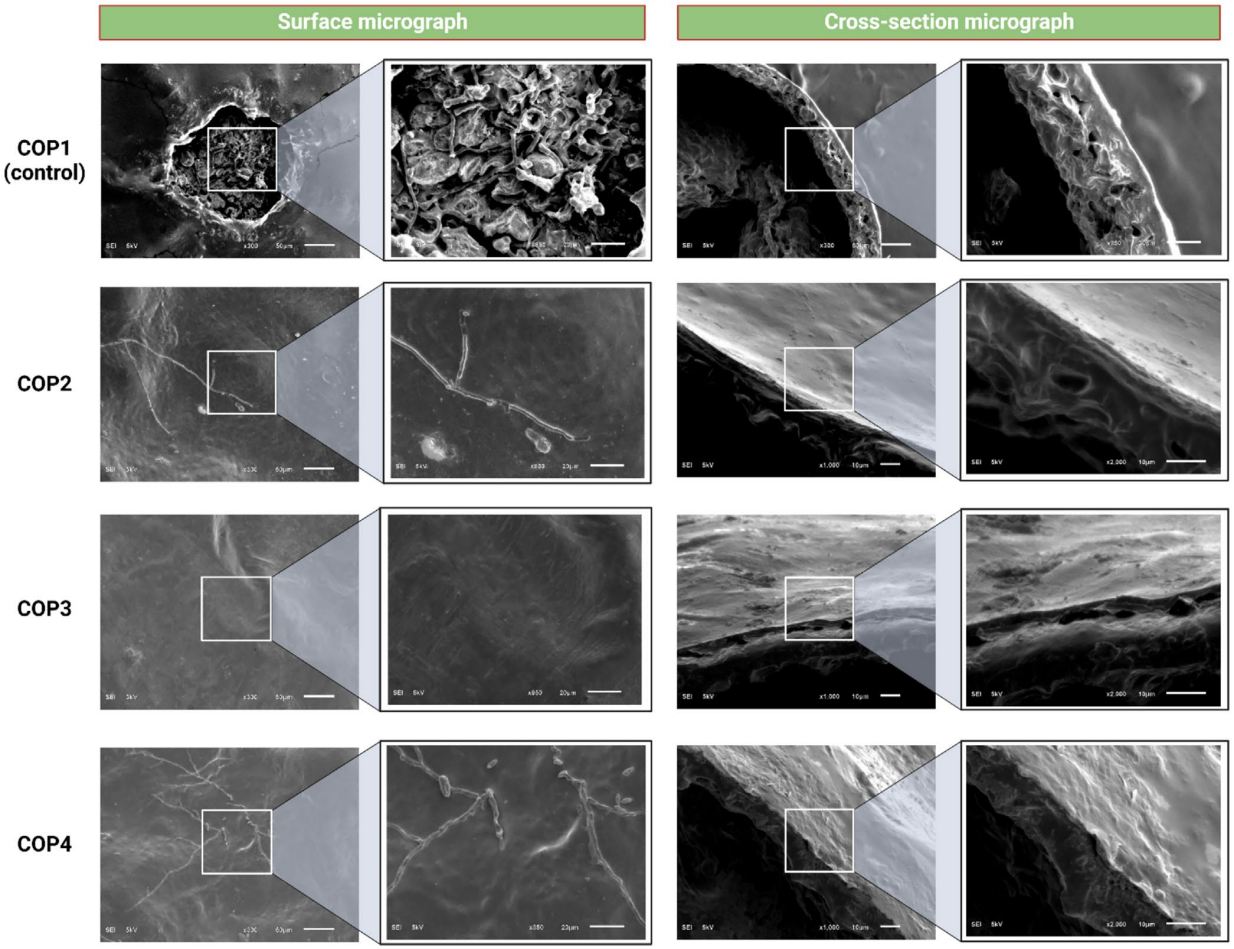
Micrographs of the fruit samples (COP1‐COP4) revealing both surface and cross‐sectional morphology, illustrating the pear surface with and without the applied coating.

The surface and cross‐sectional microstructures of the samples impact the optical and release properties, as well as oxygen and water vapor permeability (Yan et al. 2020). As displayed in Figure 4, the uncoated fruit sample (control) showed uneven and rough surface with large pores and holes, while the treated pear samples represented relatively smooth and continuous surface microstructures, especially those coated with 2% w/v chitosan and 1% v/v OEO, i.e., COP3. However, few wrinkles were observed on the surface of COP2‐ and COP4‐coated pear fruits. These findings were in close agreement with the results of Song et al. (2022), who reported that the incorporation of pine needle EO could promote uniform and dense chitosan‐based coatings. This indicates a very fine dispersion and stabilization of OEO in the chitosan matrix even after drying, suggesting a high compatibility between these two components (Elshamy et al. 2021; Stoleru et al. 2021).

**TABLE 3:** Sensory properties of pear fruits stored for 14 days with different treatments.

Sample name	Appearance	Texture	Flavor	Mouthfeel	Aroma	Overall acceptability
Day 1						
COP1	7.1 ± 1.59^aA^	6.9 ± 1.72^aA^	7.3 ± 1.41^aA^	7.2 ± 1.54^aA^	7.3 ± 1.41^aA^	7.8 ± 1.03^aA^
COP2	7.7 ± 1.41^aA^	7.7 ± 1.16^aA^	7.4 ± 1.35^aA^	7.4 ± 1.07^aA^	7.4 ± 1.17^aA^	7.9 ± 0.87^aA^
COP3	7.2 ± 1.31^aA^	7.8 ± 1.13^aA^	7.6 ± 1.50^aA^	8.1 ± 0.56^aA^	7.8 ± 1.13^aA^	7.7 ± 0.94^aA^
COP4	7.8 ± 1.47^aA^	7.5 ± 1.35^aA^	7.5 ± 1.58^aA^	7.7 ± 0.94^aA^	7.8 ± 1.22^aA^	8 ± 1.05^aA^
Day 7						
COP1	6.3 ± 2.11^bA^	5 ± 2.98^bA^	5.4 ± 1.89^bB^	5.4 ± 1.89^bB^	6.1 ± 1.44^bAB^	5.7 ± 2.45^bB^
COP2	6.9 ± 1.44^abA^	6.2 ± 1.39^baB^	6.7 ± 1.05^aAB^	6.7 ± 1.33^abAB^	6.8 ± 1.03^abAB^	7.1 ± 1.19^abA^
COP3	7.4 ± 1.43^abA^	7.2 ± 1.47^aA^	6.5 ± 1.08^abA^	6.2 ± 1.81^abB^	6.9 ± 0.87^abAB^	7.3 ± 1.05^aA^
COP4	7.8 ± 1.22^aA^	7.7 ± 1.33^aA^	7.3 ± 1.25^aA^	7.4 ± 1.71^aA^	7.3 ± 0.82^aA^	7.7 ± 1.16^aA^
Day 14						
COP1	5.7 ± 2.05^bA^	4.8 ± 2.48^bA^	4.6 ± 1.50^bB^	4.7 ± 1.25^bB^	5.5 ± 1.65^bB^	5.4 ± 1.95^bB^
COP2	6.8 ± 1.61^abA^	6.1 ± 1.44^abB^	6.3 ± 0.94^aB^	6.1 ± 1.28^abB^	6.3 ± 1.05^abB^	7 ± 1.05^aA^
COP3	7.4 ± 1.43^aA^	7.1 ± 1.37^aA^	7 ± 1.24^aA^	6.7 ± 1.76^aB^	6.7 ± 1.05^aB^	7 ± 1.24^aA^
COP4	6.8 ± 1.12^abA^	7 ± 1.56^aA^	6.9 ± 1.66^aA^	6.7 ± 1.94^aA^	7.2 ± 1.22^aA^	7.3 ± 1.33^aA^

*Note:* Values in the same column followed by different letters are significantly different. Values (A–C) show significant differences between the days for the same sample. COP1: control sample (pears without any coating); COP2: pears coated with 2% w/v chitosan; COP3: pears coated with 2% w/v chitosan and 1% v/v orange essential oil; COP4: pears coated with 2% w/v chitosan, 1% v/v orange essential oil and 8 g KMnO_4_.

### 3.7 Contact Angle Measurements

**FIGURE 5:**
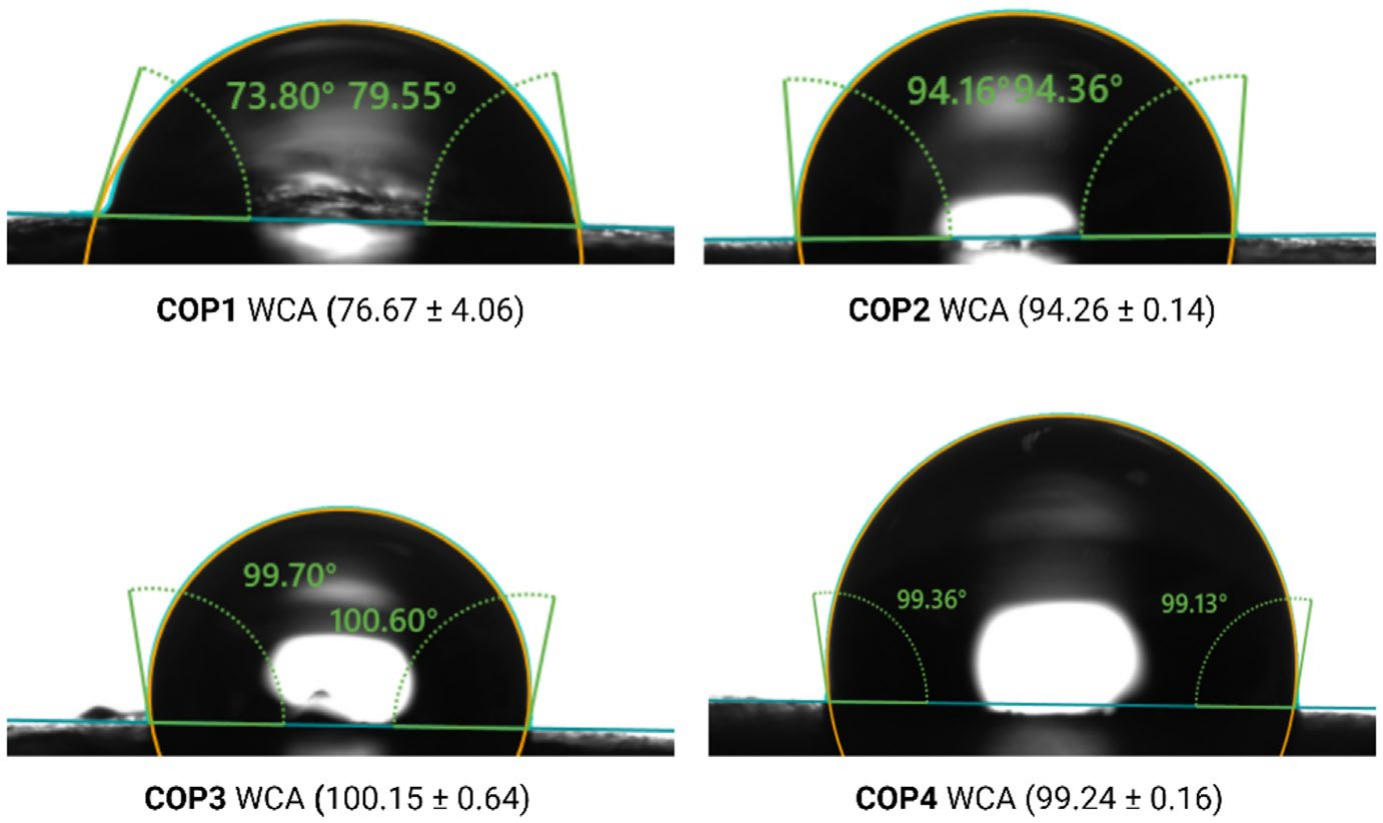
Water contact angle (WCA) of the uncoated/coated fruit samples (COP1‐COP4).

During fruit storage, it is important to ensure that the packaging material maintains its integrity and functionality even when exposed to moisture or liquids, for preserving the quality and safety of the packaged food products. To address this issue, the water resistance properties of coating materials are usually measured using water contact angle (WCA) analysis, with hydrophobic coatings having WCA values higher than 90° (Adibi et al. 2023). In general, a weaker interaction with the water droplet results in a larger WCA with the surface, meaning that the structure is more hydrophobic. This phenomenon reveals the ability of chitosan and OEO to prevent the transfer of water molecules on the fruit surface, which can be attributed to the presence of high amounts of hydrophobic groups in the chitosan structure and/or due to the hydrophobic nature of OEO (Gasti et al. 2022; Zhang et al. 2023b). On the other hand, the hydrophobicity of the fruit surface slightly decreased from 100.15° ± 0.64° to 99.24° ± 0.16° with the addition of KMnO_4_ salt (Figure 5). The obtained results are consistent with previous studies, which showed that the dense microstructure can increase the WCA of the samples (Sun et al. 2021). However, it should be noted that there was a slight difference between the WCA of COP2 and COP3, and both showed acceptable surface wettability properties for their further food applications.

### 3.8 Sensory Analysis of the Fruits

The sensory analysis of pears subjected to different coating treatments was evaluated over a 14‐day storage period, with assessments conducted on days 1, 7, and 14 (Table 3). The sensory attributes considered in our study were appearance, texture, flavor, mouthfeel, aroma, and overall acceptability, rated on a 9‐point hedonic scale. On day 1, all samples displayed high initial acceptability, with scores generally above 7.0 for all attributes. By day 7, the sensory attributes of the control group (COP1) deteriorated significantly, particularly in texture (5 ± 2.98) and overall acceptability (5.7 ± 2.45). The coated samples, especially COP4, maintained better quality. COP4 showed superior performance in appearance (7.8 ± 1.22), texture (7.7 ± 1.33), and overall acceptability (7.7 ± 1.16), demonstrating the effectiveness of the chitosan and OEO coating combined with the ethylene scavenger in preserving sensory quality. On day 14, the decline in sensory quality was more pronounced in all groups, but the control group (COP1) experienced the most significant reduction, with score values reduced to 5.7 ± 2.05 in appearance and 5.4 ± 1.95 in overall acceptability. The coated groups, particularly COP3 and COP4, maintained relatively higher scores. COP3, with chitosan and OEO coating, had scores of 7.4 ± 1.43 for appearance and 7 ± 1.24 for overall acceptability. COP4 indicated that the combination of chitosan, OEO, and the ethylene scavenger effectively preserved the sensory qualities of the pears over the 14 days.

The results suggest that chitosan coatings, especially when combined with essential oils like oregano and ethylene scavengers, can significantly enhance the shelf‐life and sensory attributes of fruits. This finding aligns with previous studies demonstrating the efficacy of chitosan‐based coatings in preserving fruit quality during storage (Aloui et al. 2017; Jiang et al. 2012; Noori and Hossaeini Marashi 2023). The inclusion of essential oils provides additional antimicrobial and antioxidant properties, further enhancing the protective effect.

## 4 Conclusions

The proposed methodology effectively reduced weight loss, the incidence of superficial scald, and improvement in the barrier properties as compared to the control group. Furthermore, the treated pears presented higher levels of total phenolics, total flavonoids, and antioxidant activity. This technique not only preserves the desirable attributes of the pears but also enhances their health‐promoting properties. Therefore, chitosan coatings, along with the presence of OEO and KMnO_4_, can effectively enhance the quality and nutritional characteristics of pear fruits during the 14 days of storage, indicating their promising role in food preservation. These findings can also help to reduce the application of synthetic antioxidants, such as butylated hydroxyanisole (BHA) and butylated hydroxytoluene (BHT) in food packaging materials, which can ultimately benefits both consumers and food manufacturers.

## Author Contributions

**Yasir Abbas Shah:** conceptualization (equal), methodology (equal), software (equal), writing – original draft (equal), writing – review and editing (equal). **Saurabh Bhatia:** conceptualization (equal), supervision (equal), writing – original draft (equal), writing – review and editing (equal). **Ahmed Al‐Harrasi:** project administration (equal), supervision (equal). **Mohammad Tarahi:** software (equal), writing – original draft (equal), writing – review and editing (equal). **Mahbubur Rahman Khan:** writing – review and editing (equal).

## Conflicts of Interest

The authors declare no conflicts of interest.

## Data Availability

Even though adequate data have been given in the form of tables and figures, however, all authors declare that if more data are required, then the data will be provided on a request basis.
